# Rehabilitation of gait after stroke: a review towards a top-down approach

**DOI:** 10.1186/1743-0003-8-66

**Published:** 2011-12-13

**Authors:** Juan-Manuel Belda-Lois, Silvia Mena-del Horno, Ignacio Bermejo-Bosch, Juan C Moreno, José L Pons, Dario Farina, Marco Iosa, Marco Molinari, Federica Tamburella, Ander Ramos, Andrea Caria, Teodoro Solis-Escalante, Clemens Brunner, Massimiliano Rea

**Affiliations:** 1 Instituto de Biomecánica de Valencia, Universitat Politécnica de Valencia, Camino de Vera, s/n ed. 9C, E46022 Valencia, Spain; 2 Grupo de Tecnología Sanitaria del IBV, CIBER de Bioingeniería, Biomateriales y Nanomedicina (CIBER-BBN). Valencia, Spain; 3 Bioengineering Group, Center for Automation and Robotics, Spanish National Research Council (CSIC). Madrid, Spain; 4 Department of Neurorehabilitation Engineering, Bernstein Center for Computational Neuroscience University Medical Center Göttingen Georg-August University. Göttingen, Germany; 5 Fondazione Santa Lucia. Roma, Italy; 6 University of Tübingen. Tübingen, Germany; 7 TECNALIA Research and Innovation Germany. Tübingen, Germany; 8 Graz University of Technology. Austria

## Abstract

This document provides a review of the techniques and therapies used in gait rehabilitation after stroke. It also examines the possible benefits of including assistive robotic devices and brain-computer interfaces in this field, according to a top-down approach, in which rehabilitation is driven by neural plasticity.

The methods reviewed comprise classical gait rehabilitation techniques (neurophysiological and motor learning approaches), functional electrical stimulation (FES), robotic devices, and brain-computer interfaces (BCI).

From the analysis of these approaches, we can draw the following conclusions. Regarding classical rehabilitation techniques, there is insufficient evidence to state that a particular approach is more effective in promoting gait recovery than other. Combination of different rehabilitation strategies seems to be more effective than over-ground gait training alone. Robotic devices need further research to show their suitability for walking training and their effects on over-ground gait. The use of FES combined with different walking retraining strategies has shown to result in improvements in hemiplegic gait. Reports on non-invasive BCIs for stroke recovery are limited to the rehabilitation of upper limbs; however, some works suggest that there might be a common mechanism which influences upper and lower limb recovery simultaneously, independently of the limb chosen for the rehabilitation therapy. Functional near infrared spectroscopy (fNIRS) enables researchers to detect signals from specific regions of the cortex during performance of motor activities for the development of future BCIs. Future research would make possible to analyze the impact of rehabilitation on brain plasticity, in order to adapt treatment resources to meet the needs of each patient and to optimize the recovery process.

## Introduction

Stroke is one of the principal causes of morbidity and mortality in adults in the developed world and the leading cause of disability in all industrialized countries. Stroke incidence is approximately one million per year in the European Union and survivors can suffer several neurological deficits or impairments, such as hemiparesis, communication disorders, cognitive deficits or disorders in visuo-spatial perception [1,2].

These impairments have an important impact in patient's life and considerable costs for health and social services [3]. Moreover, after completing standard rehabilitation, approximately 50%-60% of stroke patients still experience some degree of motor impairment, and approximately 50% are at least partly dependent in activities-of-daily-living (ADL) [4].

Hemiplegia is one of the most common impairments after stroke and contributes significantly to reduce gait performance. Although the majority of stroke patients achieve an independent gait, many do not reach a walking level that enable them to perform all their daily activities [5]. Gait recovery is a major objective in the rehabilitation program for stroke patients. Therefore, for many decades, hemiplegic gait has been the object of study for the development of methods for gait analysis and rehabilitation [6].

Traditional approaches towards rehabilitation can be qualified as bottom-up approaches: they act on the distal physical level (bottom) aiming at influencing the neural system (top), being able to rehabilitate the patients due to the mechanisms of neural plasticity. How these mechanisms are established is still unkown, despite existing several hypotheses that lead to the description of several physical therapies. Recently some authors [7] argue about new hypothesis based on the results coming from robotic rehabilitation.

An increasing number of researchers are pursuing a top-down approach, consisting on defining the rehabilitation therapies based on the state of the brain after stroke. This paper aims at providing an integrative view of the top-down approaches and their relationships with the traditional bottom-up in gait recovery after stroke. Besides, the article aim at examining how an integrative approach incorporating assistive robotic devices and brain-computer interfaces (BCI) can contribute to this new paradigm.

According to the aim of this review, this document is organized as follows. First, we cover the neurophysiology of gait, focusing on the recent ideas on the relation among cortical brain stem and spinal centers for gait control. Then, we review classic gait rehabilitation techniques, including neurophysiological and motor learning approaches. Next, we present current methods that would be useful in a top-down approach. These are assistive robotic devices, functional electrical stimulation (FES), and non-invasive BCIs based on the electroencephalogram (EEG) and functional near infrared spectroscopy (fNIRS). Finally, we present our conclusions and future work towards a top-down approach for gait rehabilitation.

Subsequently this paper is structured as follows: First there is an introduction to the physiology of gait. Then there is a review of current rehabilitation methodologies, with special emphasis to robotic devices as part of either a top-down or bottom-up approaches. Finally, we review the potential use of BCIs systems as key components for restructuring current rehabilitation approaches from bottom-up to top-down.

## Neurophysiology of gait

Locomotion results from intricate dynamic interactions between a central program and feedback mechanisms. The central program relies fundamentally on a genetically determined spinal circuit capable of generating the basic locomotion pattern and on various descending pathways that can trigger, stop and steer locomotion. The feedback originates from muscles and skin afferents as well as some senses (vision, audition, vestibular) that dynamically adapt the locomotion pattern to the requirements of the environment [8]. For instance, propioceptive inputs can adjust timing and the degree of activity of the muscles to the speed of locomotion. Similarly, skin afferents participate predominantly in the correction of limb and foot placement during stance and stimulation of descending pathways may affect locomotion pattern in specific phases of step cycle [8]. The mechanism of gait control should be clearly understood, only through a thorough understanding of normal as well as pathological pattern it is possible to maximize recovery of gait related functions in patients.

In post-stroke patients, the function of cerebral cortex becomes impaired, while that of the spinal cord is preserved. Hence, the ability to generate information of the spinal cord required for walking can be utilized through specific movements to reorganize the cortex for walking [9]. The dysfunction is typically manifested by a pronounced asymmetrical deficits [10]. Post-stroke gait dysfunction is among the most investigated neurological gait disorders and is one of the major goals in post-stroke rehabilitation [11]. Thus, the complex interactions of the neuromusculoskeletal system should be considered when selecting and developing treatment methods that should act on the underlying pathomechanisms causing the disturbances [9].

The basic motor pattern for stepping is generated in the spinal cord, while fine control of walking involves various brain regions, including cerebral motor cortex, cerebellum, and brain stem [12]. The spinal cord is found to have Central Pattern Generators (CPGs) that in highly influential definition proposed by Grillner [13] are networks of nerve cells that generate movements and enclose the information necessary to activate different motor neurons in the suitable sequence and intensity to generate motor patterns. These networks have been proposed to be "innate" although "adapted and perfected by experience". The three key principles that characterize CPGs are the following: (I) the capacity to generate intrinsic pattern of rhythmic activity independently of sensory inputs; (II) the presence of a developmentally defined neuronal circuit; (III) the presence of modulatory influences from central and peripheral inputs.

Recent work has stressed the importance of peripheral sensory information [14] and descending inputs from motor cortex [15] in shaping CPG function and particularly in guiding postlesional plasticity mechanisms. In fact for over-ground walking a spinal pattern generator does not appear to be sufficient. Supraspinal control is needed to provide both the drive for locomotion as well as the coordination to negotiate a complex environment [16].

The study of brain control over gait mechanisms has been hampered by the differences between humans and other mammals in the effects on gait of lesioning supraspinal motor centers. It is common knowledge that brain lesions profoundly affect gait in humans [17]. Therefore, it has been argued that central mechanisms play a greater role in gait control mechanisms in humans as compared to other mammals and thus data from experimental animal models are of little value in addressing central mechanisms in human locomotion [14]. One way to understand interrelationships between spinal and supraspinal centers is to analyze gait development in humans. Human infants exhibit stepping behaviour even before birth thus well before cortical descending fibers are myelinated. Infant stepping has been considered to show many of the characteristics of adult walking, like alternate legs stepping, reciprocal flexors, and extensors activation. However, it also differs from adult gait in many key features. One of the most striking differences is the capacity of CPG networks to operate independently for each leg [18]. In synthesis, there is general consensus that an innate template of stepping is present at birth [19,20] and subsequently it is modulated by superimposition of peripheral as well as supraspinal additional patterns [14].

There is also increasing evidence that the motor cortex and possibly other descending input is critical for functional walking in humans: in adults the role of supraspinal centers on gait parameters has been studied mainly by magnetic or electric transcranial stimulation (TMS) [21,22], by electroencephalography (EEG) [23] or by frequency and time-domain analyses of muscle activity (electromyography, EMG) during gait [24]. Results from these two different approaches (TMS and EMG coherence analysis) suggest that improvements in walking are associated with strengthening of descending input from the brain. Also, motor evoked potentials (MEPs) in plantar- and dorsi-flexors evoked by TMS are evident only during phases of the gait cycle where a particular muscle is active; for example, MEPs in the soleus are present during stance and absent during swing [25,26]. It is intriguing also that one of the most common problems in walking after injury to motor areas of the brain is dorsiflexion of the ankle joint in the swing phase [27]. This observation suggests that dorsiflexion of the ankle in walking requires participation of the brain, a finding that is consistent with TMS studies showing areas in the motor cortex controlling ankle dorsiflexors to be especially excitable during walking. It is also consistent with the observation that babies with immature input from the brain to the spinal cord show toe drag in walking [28]. Perhaps recovery of the ability to dorsiflexion the ankle is especially dependent on input from the motor cortex. Both line of evidence, although suggesting cortical involvement in gait control, did not provide sufficient information to provide a clear frame of cortico-spinal interplay [14].

Several research areas have provided indirect evidence of cortical involvement in human locomotion. Positron emission tomography (PET) and functional magnetic resonance imaging (fMRI) have demonstrated that during rhythmic foot or leg movements the primary motor cortex is activated, consistent with expected somatotopy, and that during movement preparation and anticipation frontal and association areas are activated [29]. Furthermore, electrophysiological studies of similar tasks have demonstrated lower limb movement related electrocortical potentials [30], as well as coherence between electromyographic and electroencephalographic signals [31].

Alexander et al. [32], by analyzing brain lesion locations in relation to post-stroke gait characteristics in 37 chronic ambulatory stroke patients suggested that damage to the posterolateral putamen was associated with temporal gait asymmetry.

In closing, gait, as simple as it might seem, is the result of very complex interactions and not at all sustained by an independent automatic machine that can be simply turn off and on [24]. The spinal cord generates human walking, and the cerebral cortex makes a significant contribution in relation to voluntary changes of the gait pattern. Such contributions are the basis for the unique walking pattern in humans. The resultant neural information generated at the spinal cord and processed at the cerebral cortex, filters through the meticulously designed musculoskeletal system. The movements required for walking are then produced and modulated in response to the environment.

Despite the exact role of the motor cortex in control of gait is unclear, available evidence may be applied to gait rehabilitation of post-stroke patients.

## Gait rehabilitation after stroke

Restoring functions after stroke is a complex process involving spontaneous recovery and the effects of therapeutic interventions. In fact, some interaction between the stage of motor recovery and the therapeutic intervention must be noticed [33].

The primary goals of people with stroke include being able to walk independently and to manage to perform daily activities [34]. Consistently, rehabilitation programs for stroke patients mainly focus on gait training, at least for sub-acute patients [35].

Several general principles underpin the process of stroke rehabilitation. Good rehabilitation outcome seems to be strongly associated with high degree of motivation and engagement of the patient and his/her family [36]. Setting goals according to specific rehabilitation aims of an individual might improve the outcomes [36]. In addition, cognitive function is importantly related to successful rehabilitation [37]. At this respect, attention is a key factor for rehabilitation in stroke survivors as poorer attention performances are associated with a more negative impact of stroke disability on daily functioning [37].

Furthermore, learning skills and theories of motor control are crucial for rehabilitation interventions. Motor adaptation and learning are two processes fundamental to flexibility of human motor control [38]. According to Martin et al., adaptation is defined as the modification of a movement from a trail-to-trial based on error feedback [39] while learning is the basic mechanism of behavioural adaptation [40]. So the motor adaptation calibrates movement for novel demands, and repeated adaptations can lead to learning a new motor calibration. An essential prerequisite for learning is the recognition of the discrepancy between actual and expected outcomes during error-driven learning [40]. Cerebral damage can slow the adaptation of reaching movements but does not abolish this process [41]. That might reflect an important method to alter certain patients' movement patterns on a more permanent basis [38].

### Classic gait rehabilitation techniques

At present, gait rehabilitation is largely based on physical therapy interventions with robotic approach still only marginally employed. The different physical therapies all aim to improve functional ambulation mostly favouring over ground gait training. Beside the specific technique used all approaches require specifically designed preparatory exercises, physical therapist's observation and direct manipulation of the lower limbs position during gait over a regular surface, followed by assisted walking practice over ground.

According to the theoretical principles of reference that have been the object of a Cochrane review in 2007 [42], neurological gait rehabilitation techniques can be classified in two main categories: neurophysiological and motor learning.

#### Neurophysiological techniques

The neurophysiological knowledge of gait principles is the general framework of this group of theories. The physiotherapist supports the correct patient's movement patterns, acting as problem solver and decision maker so the patient beings a relatively passive recipient [43]. Within this general approach according to different neurophysiological hypothesis various techniques have been proposed. The most commonly used in gait rehabilitation are summarized in the following:

♦ Bobath [44] is the most widely accepted treatment concept in Europe [45]. It hypothesizes a relationship between spasticity and movement, considering muscle weakness due to the opposition of spastic antagonists [46,47]. This method consists on trying to inhibit increased muscle tone (spasticity) by passive mobilization associated with tactile and proprioceptive stimuli. Accordingly, during exercise, pathologic synergies or reflex activities are not stimulated. This approach starts from the trunk and the scapular and pelvic waists and then it progresses to more distal segments [1,48].

♦ The Brunnström method [49] is also well known but its practice is less common. Contrary to the Bobath strategy, this approach enhances pathologic synergies in order to obtain a normal movement pattern and encourages return of voluntary movement through reflex facilitation and sensory stimulation [48].

♦ Proprioceptive neuromuscular facilitation (PNF) [50,48] is widely recognized and used but it is rarely applied for stroke rehabilitation. It is based on spiral and diagonal patterns of movements through the application of a variety of stimuli (visual, auditory, proprioceptive...) to achieve normalized movements increasing recruitments of additional motor units maximising the motor response required [51].

♦ The Vojta method [52] has been mainly developed to treat children with birth related brain damage. The reference principle is to stimulate nerves endings at specific body key points to promote the development of physiological movement patterns [53,54]. This approach is based on the activation of "innate, stored movement patterns" that are then "exported" as coordinated movements to trunk and extremities muscles. Vojta method meets well central pattern generator theories for postural and gait control and it is also applied in adult stroke patients on the assumption that brain damage somehow inhibits without disrupting the stored movement patterns.

♦ The Rood technique [55] focuses on the developmental sequence of recovery (from basic to complex) and the use of peripheral input (sensory stimulation) to facilitate movement and postural responses in the same automatic way as they normally occur.

♦ The Johnstone method [56] assumes that damaged reflex mechanisms responsible for spasticity are the leading cause of posture and movement impairment. These pathological reflexes can be controlled through positioning and splinting to inhibit abnormal patterns and controlling tone in order to restore central control. In this line at the beginning gross motor performances are trained and only subsequently more skilled movements are addressed.

#### Motor learning techniques

Just opposite to the passive role of patients implied in neurophysiological techniques, motor learning approaches stress active patient involvement [57]. Thus patient collaboration is a prerequisite and neuropsychological evaluation is required [58,59]. This theoretical framework is implemented with the use of practice of context-specific motor tasks and related feedbacks. These exercises would promote learning motor strategies and thus support recovery [60,61]. Task-specific and context-specific training are well-accepted principles in motor learning framework, which suggests that training should target the goals that are relevant for the needs of patients [36]. Additionally, training should be given preferably in the patient's own environment (or context). Both learning rules are supported by various systematic reviews, which indicate that the effects of specific interventions generalise poorly to related tasks that are not directly trained in the programme [62-64].

The motor learning approach has been applied by different authors to develop specific methodologies:

♦ The Perfetti method [65] is widely used, especially in Italy. Schematically it is a sensory motor technique and was developed originally for controlling spasticity, especially in the arms, and subsequently applied to all stroke related impairments including gait. Perfetti rehabilitation protocols start with tactile recognition of different stimuli and evolve trough passive exploitation and manipulation of muscles and joints to active manipulation. As all motor learning based techniques, Perfetti cannot be implemented without a certain degree of cognitive preservation to allow patient's cooperation.

♦ Carr and Shepherd in their motor relearning method [66] considered different assumptions. They hypothesized that neurologically impaired subjects learn in the same way as healthy individuals, that posture and movement are interrelated and that through appropriate sensory inputs it is possible to modulate motor responses to a task. In this context instruction, explanation, feedback and participation are essential. Exercises are not based on manually imposed movements but training involves therapist practice guidance for support or demonstration, and not for providing sensory input, as for instance during Perfetti type exercises [33]. The rehabilitation protocol is initially focussed on movement components that cannot be performed, subsequently functional tasks are introduced and finally generalization of this training into activities of daily living is proposed.

♦ Conductive education or Peto method [67] focuses on coping with disability and only in a subordinate level addresses functional recovery. Specific emphasis is given to integrated approaches. Particularly characteristic is the idea that feelings of failure can produce a dysfunctional attitude, which can hamper rehabilitation. Accordingly, rehabilitation protocols are mainly focus on coping with disability in their daily life by teaching them apt strategies.

♦ The Affolter method [68] assumes that the interaction between the subject and the environment is fundamental for learning, thus perception has an essential role in the learning process. Incoming information is compared with past experience ('assimilation'), which leads to anticipatory behavior. This method has been seldom used and no data are available in the literature.

♦ Sensory integration or Ayres method [69] emphasises the role of sensory stimuli and perception in defining impairment after a brain lesion. Exercises are based on sensory feedback and repetition which are seen as important principles of motor learning.

Neurorehabilitation principles and techniques have been developed to restore neuromotor function in general, aiming at the restoration of physiological movement patterns [1]. Nevertheless, it must be recalled that the gold standard for functional recovery approaches is to tailor methods for specific pathologies and patients; however, none of the above-mentioned methods has been specifically developed for gait recovery after stroke [50]. Thus, it is not surprising that the only available Cochrane review [42] on gait rehabilitation techniques states that there is insufficient evidence to determine if any rehabilitation approach is more effective in promoting recovery of lower limbs functions following stroke, than any other approach. Furthermore, Van Pepper [70] revealed no evidence in terms of functional outcomes to support the use of neurological treatment approaches, compared with usual care regimes. To the contrary, there was moderate evidence that patients receiving conventional functional treatment regimens (i.e. traditional exercises and functional activities) needed less time to achieve their functional goals [51] or had a shorter length of stay compared with those provided with specific neurological treatment approaches, such as Bobath [47,51,71]. In addition, there is strong evidence that patients benefit from exercise programmes in which functional tasks are directly and intensively trained [70,72]. Task-oriented training can assist the natural pattern of functional recovery, which supports the view that functional recovery is driven mainly by adaptive strategies that compensate for impaired body functions [73-75]. Wevers at al., underlined in a recent review, the efficacy of task-oriented circuit class training (CCT) to improve gait and gait-related activities in patients with chronic stroke [76].

Several systematic reviews have explored whether high-intensity therapy improves recovery [77-79]. Although there are no clear guidelines for best levels of practice, the principle that increased intensive training is helpful is widely accepted [38]. Agreement is widespread that rehabilitation should begin as soon as possible after stroke, [80] and clinical trials of early commenced mobility and speech interventions are underway.

According to these data, Salbach et al [81] suggested that high-intensity task oriented practice may enhance walking competency in patients with stroke better than other methods, even in those patients in which the intervention was initiated beyond 6 months after stroke. In contrast, impairment focused programmes such as muscle strengthening, muscular re-education with support of biofeedback, neuromuscular or transcutaneous nerve stimulation showed significant improvement in range of motion, muscle power and reduction in muscle tone; however these changes failed to generalize to the activities themselves [70]. Interestingly, a similar trend was found for studies designed to improve cardiovascular fitness by a cycle ergometer [82]. Interestingly, no systematic review has specifically addressed whether the less technologically demanding intervention of over ground gait training is effective at improving mobility in stroke patients. While there is a clinical consensus that over ground gait training is needed during the acute stage of recovery for those patients who cannot walk independently [83], there has been little discussion of whether over ground gait training would be beneficial for chronic patients with continuing mobility deficits. States et al. [84] suggested that over ground gait training, has no significant effects on walking function, although it may provide small, time-limited benefits for the more uni-dimensional variables of walking speed, Timed Up and Go test and 6 Minutes Walking Test. Instead, over ground gait training may create the most benefit in combination with other therapies or exercise protocols. This hypothesis is consistent with the finding that gait training is the most common physical therapy intervention provided to stroke patients [35]. It is also consistent with other systematic reviews that have considered the benefit of over ground gait training in combination with treadmill training or high-technology approaches like body weight support treadmill training (BWSTT) [85] or with exercise protocols in acute and chronic stroke patients [86]. This combination of rehabilitation strategies, as will be described in the next section of this paper, appear to be more effective than over ground gait training alone, perhaps because they require larger amounts of practice on a single task than is generally available within over ground gait training.

### Robotic devices

Conventional gait training does not restore a normal gait pattern in the majority of stroke patients [87]. Robotic devices are increasingly accepted among many researchers and clinicians and are being used in rehabilitation of physical impairments in both the upper and lower limbs [88,89].

These devices provide safe, intensive and task-oriented rehabilitation to people with mild to severe motor impairments after neurologic injury [90]. In principle, robotic training could increase the intensity of therapy with quite affordable costs, and offer advantages such as: *i*) precisely controllable assistance or resistance during movements, *ii*) good repeatability, *iii*) objective and quantifiable measures of subject performance, *iv*) increased training motivation through the use of interactive (bio)feedback. In addition, this approach reduces the amount of physical assistance required to walk reducing health care costs [88,91] and provides kinematic and kinetic data in order to control and quantify the intensity of practice, measure changes and assess motor impairments with better sensitivity and reliability than standard clinical scales [88,90,92].

Because of robotic rehabilitation is intensive, repetitive and task-oriented, it is generally in accordance with the motor re-learning program [36,63], more than with the other rehabilitative approaches reported above in this document.

The efficacy of the human-robot interactions that promote learning depends on the actions either imposed or self-selected by the user. The applied strategies with available robotic trainers aim at promoting effort and self initiated movements. The control approaches are intended to i) allow a margin of error around a target path without providing assistance, ii) trigger the assistance in relation to the amount of exerted force or velocity, iii) enable a compliance at level of the joint and iv) detrend the robotic assistance by means of what has been proposed as a forgetting factor. In the former approach, the assumption is that the human resists applied forces by internally modelling the force and counteracting to it.

Regarding current assistance strategies employed in robotic systems, the assist-as-needed control concept has emerged to encourage the active motion of the patient. In this concept, the goal of the robotic device is to either assist or correct the movements of the user. This approach is intended to manage simultaneous activation of efferent motor pathways and afferent sensory pathways during training. Current assist-as-needed strategies face one crucial challenge: the adequate definition of the desired limb trajectories regarding space and time the robot must generate to assist the user during the exercise. Supervised learning approaches that pre-determine reference trajectories have been proposed to this purpose. Assist-as-needed approach has been applied as control strategy for walking rehabilitation in order to adapt the robotic device to varying gait patterns and levels of support by means of implementing control of mechanical impedance. Zero-impedance control mode has been proposed to allow free movement of the segments. Such approach, referred to as "path control" has been proposed with the Lokomat orthosis, (Hocoma, AG; Switzerland) [93] resulting in more active EMG recruitments when tested with spinal cord injury subjects. The concept of a virtual tunnel that allows a range of free movement has been evaluated with stroke patients in the lower limb exoskeleton ALEX [94].

Regarding rehabilitation strategies, the most common robotic devices for gait restoration are based on task-specific repetitive movements which have been shown to improve muscular strength, movement coordination and locomotor retraining in neurological impaired patients [95,96]. Robotic systems for gait recovery have been designed as simple electromechanical aids for walking, such as the treadmill with body weight support (BWS) [97], as end-effectors, such as the Gait Trainer (Reha-Technologies, Germany, GT)[98], or as electromechanical exoskeletons, such as the Lokomat [99]. On treadmills, only the percentage of BWS and walking speed can be selected, whereas on the Lokomat, the rehabilitation team can even decide the type of guidance and the proper joint kinematics of the patients' lower limbs. On the other hand, end effector devices lie between these two extremes, including a system for BWS and a controller of end-point (feet) trajectories.

A fundamental aspect of these devices is hence the presence of an electromechanical system for the BWS that permits a greater number of steps within a training session than conventional therapy, in which body weight is manually supported by the therapists and/or a walker [100,101]. This technique consists on using a suspension system with a harness to provide a symmetrical removal of a percentage of the patient's body weight as he/she walks on a treadmill or while the device moves or support the patient to move his/her lower limbs. This alternative facilitates walking in patients with neurological injuries who are normally unable to cope with bearing full weight and is usually used in stroke rehabilitation allowing the beginning of gait training in early stages of the recovery process [102].

However, some end-effector devices, such as the Gait Trainer, imposes the movements of the patient' feet, mainly in accordance to a bottom-up approach similar to the passive mobilizations of Bobath method [38] instead of a top-down approach. In fact, a top-down approach should be based on some essential elements for an effective rehabilitation such as an active participation [37], learning skills [38] and error-drive-learning [39].

Several studies support that retraining gait with robotic devices leads to a more successful recovery of ambulation with respect to over ground walking speed and endurance, functional balance, lower-limb motor recovery and other important gait characteristics, such as symmetry, stride length and double stance time [96,91,103].

In these studies, BWS treadmill therapy has sometimes been associated, from a clinical point of view, to the robotic therapies, even if treadmill should not be considered as a robot for their substantial engineering differences. In fact, in a recent Cochrane, electromechanical devices were defined as any device with an electromechanical solution designed to assist stepping cycles by supporting body weight and automating the walking therapy process in patients after stroke, including any mechanical or computerized device designed to improve walking function and excluding only non-weight-bearing devices [104].

*Visintin et al *[105] reported that treadmill therapy with BWS was more effective than without BWS in subacute, nonambulatory stroke patients, as well as showing advantages over conventional gait training with respect to cardiovascular fitness and walking ability.

*Luft et al *[106] compared the effects of 6-month treadmill training versus comparable duration stretching on walking, aerobic fitness and in a subset on brain activation measured by functional MRI. The results suggested that treadmill training promotes gait recovery and fitness, and provides evidence of neuroplasticity mechanisms.

*Mayr et al *[107] found more improvement during the Lokomat training phase than during the conventional physical therapy phase after a rehabilitation program that applied these two different techniques for gait training.

On the other hand, *Peshkin et al *[95] attempted to identify users and therapists' needs through observations and interviews in rehabilitation settings to develop a new robotic device for gait retraining in over-ground contexts. They intended to establish key tasks and assess the kinematics required to support those tasks with the robotic device making the system able to engage intense, locomotor-specific, BWS training over ground while performing functional tasks.

As most complex robots need to be permanently installed in a room, patients have to be moved from their beds to attend the rehabilitation. This is the main reason why therapy cannot be provided as soon as possible after stroke. In order to overcome this limitation, a robotic platform was developed by Monaco et al [108,109] that consists of providing leg manipulation, with joint trajectories comparable with those related to natural walking for bedridden patients.

On the other hand, robotic feedback training is an emerging but promising trend to constitute an active rehabilitation approach and novel methods to evaluate motor function. Forrester et al [110] tested the robotic feedback approach in joint mobilization training, providing assistance as needed and allowing stroke patients to reach targets unassisted if they are able. Song et al [111] investigated the effect of providing continuous assistance in extension torque with a controlled robotic system to assist upper limb training in patients with stroke. The results suggested improved upper limb functions after a twenty-session rehabilitation program. Ueda et al [112] tested a computational algorithm that computes control commands (muscle force prediction) to apply target muscle forces with an exoskeleton robot. The authors foresee its application to induce specific muscle activation patterns in patients for therapeutic intervention.

Huang et al [113] assessed with an exoskeleton the amount of volitional control of joint torque and its relation to a specific function post injury, e.g. when rehabilitation involves the practice of joint mobilization exercises.

However, other studies have provided conflicting results regarding the effectiveness of robotic devices for ambulatory and/or chronic patients with stroke [114,115]. A recently updated Cochrane review [104] has demonstrated that the use of electromechanical devices for gait rehabilitation increases the likelihood of walking independently in patients with subacute stroke (odd ratio = 2.56) but not in patients with chronic stroke (odd ratio = 0.63). Furthermore, some other problems are still limiting a wider diffusion of robotic devices for gait restoring, such as their high costs and the skepticism of some members of rehabilitation teams [116] probably based on the lacks of clear guidelines about robotic training protocols tailored on patients' motor capacity [117].

More recently, Morone et al [118] have proposed to change the scientific question about the effectiveness of these robotic devices into "who may benefit from robotic-assisted gait training?". The authors found that robotic therapy combined with conventional therapy is more effective than conventional therapy alone in severely affected patients.

At the light of all the above studies, the efficacy of each robotic device in neurorehabilitation seems to be related to a correct identification of the target population, in accordance with a generalization of the assist-as-needed strategy. Furthermore, it seems clear that a deeper knowledge about the proper selection of robotic devices, their training parameters and their effects on over ground walking performance for each patient can surely increase awareness of the potentialities of robotic devices for walking training in rehabilitation [117]. It is hence conceivable to conclude that more constraining devices, such as Lokomat, could be helpful at the beginning of rehabilitation and with more severely affected patients, whereas end-effector devices and then treadmill, could be more effective in more advanced stages of rehabilitation and/or in less affected patients [97].

### Functional Electrical Stimulation

Functional Electrical Stimulation (FES) is a useful methodology for the rehabilitation after stroke, along or as a part of a Neuro-robot [119].

FES consists on delivering an electric current through electrodes to the muscles. The current elicits action potentials in the peripheral nerves of axonal branches and thus generates muscle contractions [120].

FES has been used in rehabilitation of chronic hemiplegia since the 1960s.

The firsts applications of FES in stroke recovery were focused on drop-foot correction, later researchers began to selectively stimulate the muscles for dorsiflexion of the foot as well as other key muscle groups in the affected leg [121].

Stanic et al [122] found that multichannel FES, given 10 to 60 minutes, 3 times per week for 1 month, improved gait performance in hemiplegic subjects.

Bogataj et al [123] applied multichannel FES to activate lower limb muscles of chronic hemiplegic subjects. After daily treatment 5 days per week for 1 to 3 weeks, the data provided by the stride analyzer and the ground reaction measuring system, as well as observations of the subjects' gait, suggested that multichannel FES may be a suitable treatment for walking recovery.

Later studies established the beneficial effects on the gait pattern of ambulatory patients, which, however, were likely to disappear after a few months [124].

Kottink et al [125] performed a meta-analysis to verify the capability of FES to improve gait speed in subjects post-stroke. Patients were treated with FES from 3 weeks to 6 months. The authors determined that gait speed improved significantly during FES treatment (orthotic effect). Nevertheless, it was unknown whether these improvements in walking speed were maintained after the FES was removed (therapeutic effect).

On the other hand there is strong evidence that FES combined with other gait retraining strategies results in improvements in hemiplegic gait, faster rehabilitation process and enhancement of the patients' endurance [121,124,126].

Lindquist et al [11] compared the effects of using treadmill training with BWS alone and in combination with FES on gait and voluntary lower limb control of 8 ambulatory patients with chronic stroke. The combined use of these two techniques led to an enhancement in motor recovery and seemed to improve the gait pattern (stance duration, cadence and cycle length symmetry).

Maple et al [127] attempted to evaluate the effectiveness of gait training comparing 3 different therapies: over ground walking training and electromechanical gait trainer with or without FES, for 54 patients with subacute stroke. After 4 weeks of 20-minute daily sessions, the groups that performed electromechanical gait with and without FES showed better improvement in comparison to the over ground walking group.

Tong et al [128] reported improvements in several functional and clinical scales for 2 patients with acute ischemic stroke after 4 weeks of electromechanical gait training with simultaneous FES.

Both robotic devices and FES can be controlled or triggered by biological signals recorded from the patient. For example, signals recorded from muscles (electromyography, EMG) can provide information on the level of residual activation and on the neural control strategies. In these applications, the patient actively participates in intensive and repetitive task-oriented practice while task support (by robotic devices or FES) is triggered by residual myoelectric activity during volitional control. With respect to passive movements, it has been shown that motor learning is promoted by the use of residual EMG activity to trigger external devices assisting the movement [129]. The rationale for enhanced motor learning is that patients, such as people with stroke with severe paresis, would lack appropriate proprioceptive feedback due to a lesion involving sensory pathways. The use of EMG to trigger an action supported by an external device would reinstate appropriate proprioceptive feedback because the feedback is directly triggered by the voluntary movement. The neural activity associated with the specification of the goal and outcome of movement would have a causal relation and promote learning [130]. During rehabilitation, the residual myoelectric activity and thus voluntary execution of the task increases. Such positive feedback loop further enhances learning. This mechanism explains, for example, the therapeutic effect of FES. When paretic muscles are electrically stimulated in order to improve a function, better performance is observed if the stimulation is triggered by residual muscular activity compared to passive stimulation [131].

Similar mechanisms are supposed to be triggered by decoding the patient intention directly from the brain activity. This approach, which is referred to as brain-computer interfacing (BCI), requires more complex decoding methods than those based on muscular activities but provides a direct link with the neural circuitries activated during movement following the principles of a top-down approach.

## Brain-Computer Interfaces

Brain-Computer Interface (BCI) systems record, decode, and translate some measurable neurophysiological signal into an effector action or behavior [132]. Therefore, according to this definition BCIs are potentially a powerful tool for being part of a Top-Down approach for neuro-rehabilitation as far as they can record and translate useful properties of brain activity related with the state of recovery of the patients.

BCIs establish a direct link between a brain and a computer without any use of peripheral nerves or muscles [133], thereby enabling communication and control without any motor output by the user [134,135]. In a BCI system, suitable neurophysiological signals from the brain are transformed into computer commands in real-time. Depending on the nature of these signals, different recording techniques serve as input for the BCI [136-138]. Volitional control of brain activity allows for the interaction between the BCI user and the outside world.

There are several methods available to detect and measure brain signals: systems for recording electric fields (electroencephalography, EEG, electrocorticography, ECoG and intracortical recordings using single electrodes or an electrode array) or magnetic fields (magnetoencephalography, MEG), functional magnetic resonance imaging (fMRI), positron emission tomography (PET), and functional near-infrared spectroscopy (fNIRS) [139,140]. Although all these methods have already been used to develop BCIs, in this paper we focus only on the non-invasive technologies that are portable and relatively inexpensive: EEG and fNIRS. Furthermore, we review publications that envisioned the inclusion of BCI for stroke rehabilitation and the first reports on its inclusion.

In the last decades, an increasing number of BCI research groups have focused on the development of augmentative communication and control technology for people with severe neuromuscular disorders, including those neurologically impaired due to stroke [132,141,142].

Daly et al. [139] explained this expansion of the BCI research field through four factors:

• Better understanding of the characteristics and possible uses of brain signals.

• The widely recognition of activity-dependent plasticity throughout the CNS and its influence on functional outcomes of the patient.

• The growth of a wide range of powerful low-cost hardware and software programs for recording and analyzing brain signals during real-time activities.

• The enhancement of the incidence and consideration of the people with severe motor disabilities.

One of the most popular neurophysiological phenomena used in BCI research is modulation of sensorimotor rhythms through motor imagery (MI) [143]. Imagination of limb movement produces a distinctive pattern on the motor cortex that can be detected online from the EEG [144-146], MEG [147], ECoG [148-150], fMRI [151] and fNIRS [152,153].

Mental simulation of movement, engages the primary motor cortex in a similar way that motor execution does [154]. Motor imagery (MI) patterns have been found in healthy people [155-157], ALS patients [158], SCI patients [159,160], and in stroke patients [161]. Since MI does not require motor output, it can be used to "cognitively rehearse physical skills in a safe, repetitive manner" [162], even in patients with no residual motor function.

In particular, for motor recovery after stroke, MI has been extensively exploited to promote neuroplasticity in combination with traditional physiotherapy and robot-aided therapy [163]. For example, Page et al. [162] showed that including a session of MI (30 minutes) after the usual physiotherapy (twice a week during six weeks) led to a significant reduction in affected arm impairment and significant increase in daily arm function, compared to a control group with physiotherapy but without MI sessions. MI sessions were guided by an audio tape describing the movements in both visual and kinesthetic ways. It can be seen that supporting MI with a BCI, would provide an objective measure of cortical activation during the MI therapy sessions.

In an early report on BCI control by stroke patients, Birbaumer et al. [140] reported on a MEG-based BCI. Chronic stroke patients with no residual hand function were trained to produce reliable MI patterns (volitional modulations of the sensorimotor rhythms around 8--12 Hz, through imagery of hand movements) to open and close a hand orthosis. To this end, between ten and twenty training sessions were required. Once the patients were able to control the device, further therapy sessions were carried out with a portable EEG-based BCI. It was mentioned that, as a side effect, the patients experienced "complete relief of hand spasticity" but not details were provided.

After this report, other research groups presented reports on future prospects of BCIs and the role of BCIs in neurological rehabilitation.

Buch et al. [132] reported that six out of eight patients with chronic hand plegia resulting from stroke could control the MEG-BCI after 13 to 22 sessions. Their performance ranged between 65% and 90% (classification accuracy), however, none of the patients showed significant improvement in their hand function after the BCI training.

Recently, Broetz et al. [164,165] reported the case of one chronic stroke patient trained over one year with a combination of goal-directed physical therapy and the MEG/EEG-BCI reported in [132,140]. After therapy, hand and arm movement ability as well as speed and safety of gait improved significantly. Moreover, the improvement in motor function was associated with an increased MI pattern (mu oscillations)from the ipsilesional motor cortex.

According to the literature, MEG and fMRI are better at locating stroke lesions and the neural networks involved in MI, thus, making those techniques the best choice for assessing changes in the motor activity that could foster and improve motor function [133,145,140,166-169]. However, due to better portability and lower cost, EEG is a better choice for clinical setups, real time systems, and MI-based therapy, while functional methods like fNIRS are still an option. The next sections present the current approaches and the latest development in motor function recovery after stroke, using EEG-based and fNIRS-based BCIs.

### Electroencephalography-based BCIs

Nowadays, there are only a few reports of Electroencephalograpy (EEG)-based BCIs for rehabilitation in stroke patients. The major part of these reports for stroke recovery focus on the rehabilitation of upper limbs, specifically of hand movements. Moreover, most of these reports focus on BCI performance of stroke patients and only a few of them have shown a real effect of BCI usage on motor recovery. Ang et al. [170] presented a study where a group of eight hemiparetic stroke patients received twelve sessions (one hour each, three times a week during four weeks) of robotic rehabilitation guided by an EEG-BCI. If the BCI detected the patient's intention to move, a robotic device (MIT-Manus) guided the movement of the patient's hand. A control group (ten patients) received the same number of standard robotic rehabilitation sessions (passive hand movements), without BCI control. Post-treatment evaluation of hand function (Fugl Meyer scale, relative to the pre-treatment evaluation) showed a significant improvement in both groups, but no differences between them. Between subsets of participants with function improvements (six in the experimental and seven in the control group), the experimental group presented a significantly greater improvement of hand motor function after adjustment of age and gender. Based on their own previous results, Ang et al. [171] reported that 89% of chronic stroke patients (from a total sample of 54 patients) can operate an EEG-BCI with a performance greater than chance level, and that the performance is not correlated with their motor function (Fugl Meyer scale, Pearson's correlation r = 0.36).

In contrast, Platz et al. [172] found a correlation between the ability to produce a desynchronization of the sensorimotor rhythms (associated with cortical activation) and the clinical motor outcome of acute and sub-acute stroke patients.

Daly et al. [166] presented a case study where one stroke patient (ten months after stroke) was able to perform isolated index finger extension after nine sessions (45 minutes, three times a week during three weeks) of training with FES controlled by an EEG-based BCI. Before treatment, the patient was unable to produce isolated movement of any digit of her affected hand. The BCI differentiated between movement attempts and a relaxation state. The authors reported that the patient was able to modulate sensorimotor rhythms (mu band) of her ipsilesional hemisphere for attempted and imagined movement after the first session; BCI control for relaxation was achieved until the fifth session. Both control and relaxation are desirable functions of the central nervous system (CNS) that allow to improve motor function and to reduce spasms. Prior to this work, Daly et al. [173], showed post-treatment changes in the EEG of people with stroke (reduction of abnormal cognitive planning time and cognitive effort) that occurred in parallel with improvement in motor function.

Prasad et al. [174,175] presented a pilot study with five chronic stroke patients, based on the findings of Page et al. [162]. In the study, the patients completed twelve sessions of BCI training (twice a week during six weeks). The BCI detected imagery of left vs. right hand movements in real time, and translated the cortical activity into the direction of a falling ball (presented at the top of the screen). The participants could control the ball by modulating their sensorimotor rhythms to hit a target at the bottom of the screen at the left or right side. After the training, the patients' average performance ranged between 60% and 75%, but did not show any significant improvements in their motor function. These results are in line with the report of Buch et al. [132] with the combined MEG/EEG BCI training (previously described).

Tan et al. [176] reported that four out of six post-acute stroke patients (less than three months after lesion) could modulate their sensorimotor rhythms to activate FES of the wrist muscles. Such findings are important since most of the post-stroke recovery occurs during the six months following the lesion, thus traditional and robotic-aided therapy could start as early as three months, with the possible inclusion of a BCI.

There is enough evidence to support the assumption that BCIs could improve motor recovery, but there are no long term and group studies that show a clear clinical relevance.

There is also evidence that MI of lower limbs, e.g. dancing or foot sequences, helps to improve gait [177,178] and coordination of lower limb movements [179]. Moreover, Malouin et al [180] showed differences between hand and foot MI after stroke. On the other hand, some studies suggest that there is a common mechanism influencing upper and lower limb recovery simultaneously, independently of the limb chosen for the rehabilitation therapy [181,182]. While upper limb recovery is the focus of attention, lower limb and gait function have not been studied in combination with BCIs yet. Recent reports on EEG analysis during gait, suggest that it is possible to find neural correlates of gait [23] and to decode leg movement [183]. Whether EEG-BCIs, or any BCI at all, are helpful for gait rehabilitation, is still an interesting question that remains open.

### Functional near infrared spectroscopy-based BCIs

Functional near infrared spectroscopy (fNIRS) is a non-invasive psycho-physiological technique that utilizes light in the near infrared range (700 to 1000 nm) to determine cerebral oxygenation, blood flow, and metabolic status of localized regions of the brain. The degree of increase in regional cerebral blood flow (rCBF) exceeds that of increases in regional cerebral oxygen metabolic rate (rCMRO2) resulting in a decrease in deoxygenated haemoglobin (deoxyHb) in venous blood. Thus, increase in total haemoglobin and oxygenated haemoglobin (oxyHb) with a decrease in deoxygenated haemoglobin is expected to be observed in activated brain areas during fNIRS measurement. fNIRS uses multiple pairs or channels of light sources and light detectors operating at two or more discrete wavelengths. The light source is usually a light emitting diode. Three techniques are available for NIRS signal acquisition, continuous-wave spectroscopy, time-resolved spectroscopy and frequency-domain techniques [184]. Continuous-wave spectroscopy is the approach used in the majority of the neuroimaging as well as brain-computer interface (BCI) studies. In this technique, the optical parameter measured is attenuation of light intensity due to absorption by the intermediate tissue. The source and the detector are separated by a distance of 2-7 cm to allow light to pass through the intermediate layers of scalp, skull and tissue to reach the surface of the brain again. The greater the distance between the source and the detector, the greater is the chance that the near-infrared light reaches the cortical surface. However, the attenuation of light due to absorption and scattering increases with the source-detector distance. The changes in the concentration of oxyHb and deoxyHb are computed from the changes in the light intensity at different wavelengths, using the modified Beer-Lambert equation [184].

The favorable properties of the fNIRS approach are its simplicity, flexibility and high signal to noise ratio. fNIRS provides spatially specific signals at high temporal resolution and it is portable and less expensive than fMRI. Human participants can be examined under normal conditions such as sitting in a chair, without their motion being severely restricted. However, the depth of brain tissue which can be measured is only 1-3 cm, restricting its applications to the cerebral cortex. With exciting developments in portable fNIRS instruments incorporating wireless telemetry [185], it is now possible to monitor brain activity from freely moving subjects [186,187] thus enabling more dynamic experimental paradigms, clinical applications and making it suitable for implementation on BCIs.

As this paper focuses on rehabilitation of gait after stroke, the next sections will analyze the literature regarding gait performance using fNIRS and its application in stroke rehabilitation.

### Assessment of gait with fNIRS

Increasing evidence indicates that fNIRS is a valuable tool for monitoring motor brain functions in healthy subjects and patients. Less sensitivity of fNIRS to motion artifacts allows the experimenters to measure cortical hemodynamic activity in humans during dynamic tasks such as gait.

Miyai and colleagues [188] recorded cortical activation in healthy participants associated with bipedal walking on a treadmill. They reported that walking was bilaterally associated with increased levels of oxygenated and total hemoglobin in the medial primary sensorimotor cortex (SMC) and the supplementary motor area (SMA). Alternating foot movements activated similar but less broad regions. Gait imagery increased activities caudally located in the SMA.

A study from Suzuki et al [189] explored the involvement of the prefrontal cortex (PFC) and premotor cortex (PMC) in the control of human walking and running by asking participants to perform three types of locomotor tasks at different speeds using a treadmill. During the acceleration periods immediately preceded reaching the steady walking or running speed, the levels of oxyHb increased, but those of deoxyHb did not in the frontal cortices. The changes were greater at the higher locomotor speed in the bilateral PFC and the PMC, but there were less speed-associated changes in the SMC. The medial prefrontal activation was most prominent during the running task.

Similarly, Mihara and colleagues [190] reported the involvement of the PMC and PFC in adapting to increasing locomotor speed.

A recent fNIRS study [191] showed that preparation for walking cued by a verbal instruction enhanced frontal activation both during the preparation and execution of walking as well as walking performance.

Altogether the studies on healthy participants reported an association between the PFC, SMA and SMC and control of gait speed. Moreover, the involvement of the left PFC might depend on an age-related decline in gait capacity in the elderly [192].

Thus far, few studies utilized fNIRS to assess cortical activation patterns in stroke patients. Cortical activation during hemiplegic gait was assessed in six non-ambulatory patients with severe stroke, using an fNIRS imaging system [193]. Patients performed tasks of treadmill walking under partial BWS, either with mechanical assistance in swinging the paretic leg control (CON) or with a facilitation technique that enhanced swinging of the paretic leg (FT), provided by physical therapists. Gait performance was associated with increased oxyHb levels in the medial primary sensorimotor cortex in the unaffected hemisphere greater than in the affected hemisphere. Both cortical mappings and quantitative data showed that the PMC activation in the affected hemisphere was enhanced during hemiplegic gait. Moreover, cortical activations and gait performance were greater in walking with FT than with CON. In a follow-up study the same authors investigated cerebral mechanisms underlying locomotor recovery after stroke [194]. Locomotor recovery after stroke seems to be associated with improvement of asymmetry in SMC activation and enhanced PMC activation in the affected hemisphere. In particular a correlation between improvement of the asymmetrical SMC activation and improvement of gait parameters were measured.

Furthermore, Mihara and colleagues [195] compared cortical activity in patients with ataxia during gait on a treadmill after infratentorial stroke with those in healthy control subjects observed a likely compensatory sustained prefrontal activation during ataxic gait.

Overall, these studies demonstrate the suitability of fNIRS for detecting brain activity during normal and impaired locomotion and subsequently as being part of a top-down strategy for rehabilitation.

### fNIRS-BCI in stroke rehabilitation

Coyle et al. [196] and Sitaram and Hoshi et al. [197] were the first to conduct experiments to investigate the use of fNIRS for developing BCIs.

Sitaram et al [197] reported that MI produced similar but reduced activations in comparison to motor execution when participants used overt and covert finger tapping of left and right hands.

In the study by Coyle and Ward et al. [196] a BCI system provided visual feedback by means of a circle on the screen that shrunk and expanded with changes in hemoglobin concentration while participants imagined continually clenching and releasing a ball. An intensity threshold of the hemoglobin concentration from the contralateral optodes on the motor cortex was used to determine the actual brain state [196,197]. In a follow-up experiment, Coyle et al. [152] used their custom-built fNIRS instrument to demonstrate a binary switching control called the Mindswitch with the objective of establishing a binary yes or no signal for communication. The fNIRS signal used for this purpose was derived from a single channel on the left motor cortex elicited by imagined movement of the right hand. The fNRIS based Mindswitch system tested on healthy participants showed that the number of correct classifications to the total number of trials was on the average more than 80%.

Recently, several studies reported fNIRS based BCI implementations [197-201]. Sitaram et al [198,202] published the first controlled evaluation of an fNIRS-BCI. They used a continuous wave multichannel NIRS system (OMM-1000 from Shimadzu Corporation, Japan) over the motor cortex on healthy volunteers, to measure oxyHb and deoxyHb changes during left hand and right hand motor execution and imagery. The results of signal analysis indicated distinct patterns of hemodynamic responses which could be utilized in a pattern classifier towards developing a BCI. Two different pattern recognition techniques, Support Vector Machines (SVM) and Hidden Markov Model (HMM) were applied for implementing the automatic pattern classifier. SVMs are learning systems developed by Vapnik and his co-workers [203]. SVM has been demonstrated to work well in a number of real-world applications including BCI [204]. A Markov model is a finite state machine which can be used to model a time series. HMMs were first successfully applied for speech recognition, and later in molecular biology for modelling the probabilistic profile of protein families [205]. This was the first time that SVM and HMM techniques were used to classify NIRS signals for the development of a BCI. Data for finger tapping and imagery were collected in two separate sessions for all participants. The analysis showed that, typically, concentration of oxyHb increased and concentration of deoxyHb decreased during both finger tapping and motor imagery tasks. However, changes in concentration, both for oxyHb and deoxyHb, for finger tapping were greater than those for motor imagery. Furthermore, channels on the motor cortex of the contralateral hemisphere showed activation by an increase in oxyHb and decrease in deoxyHb, while the channels on the ipsilateral hemisphere either showed similar response but to a smaller extent, or in a reversed manner potentially indicating inhibition. Reconstruction of topographic images of activation showed that there exist distinct patterns of hemodynamic responses as measured by fNIRS to left hand and right hand motor imagery tasks which could be utilized in a pattern classifier towards developing a BCI. Finger tapping data were classified with better accuracy compared to motor imagery data, by both classification techniques for all the subjects. Between the two pattern classification techniques, HMM performed better than SVM, for both finger tapping and motor imagery tasks. The results of high accuracy of offline pattern classification of NIRS signals during motor imagery tasks (SVM: 87.5%, HMM: 93.4%) indicated the potential use of such techniques to the further development of BCI systems. Towards this end, it was implemented a NIRS-BCI system incorporating a word speller as a language support system for people with disabilities. The authors concluded that NIRS provides an excellent opportunity to use a variety of motor and cognitive activities to detect signals from specific regions of the cortex.

With the objective of developing a specific fNIRS-BCI for rehabilitation of patients with lower limbs impairment, Rea and colleagues [206] assessed fNIRS capability to capture specific brain activity related to motor preparation of lower limb movements. Preliminary results showed an increase of oxyHb in the parietal cortex 9 to 11 s before legs' movement onset.

Overall these findings indicate that despite the inherent latency of the hemodynamic response fNIRS provides researchers with an excellent opportunity to use motor activities to detect signals from specific regions of the cortex for the development of future BCIs.

## Conclusions

After stroke, gait recovery is a major objective in the rehabilitation program, therefore a wide range of strategies and assistive devices have been developed for this purpose. However, estimating rehabilitation effects on motor recovery is complex, due to the interaction of spontaneous recovery, whose mechanisms are still under investigation, and therapy.

The approaches used in gait rehabilitation after stroke include neurophysiological and motor learning techniques, robotic devices, FES, and BCIs.

Despite being successful, the main principles of current rehabilitative approaches remain unclear [7], and probably this is the main reason why, apparently contradictory therapies produce similar outcomes. At this respect can be notice that the majority of rehabilitative methodologies nowadays applied are bottom-up in the sense that they act on the physical level and expect for changes at the central neural system level. Therefore, it is reasonable to expect a better insight in the understanding of the rehabilitative process if top-down approaches are considered. Besides, these new insights can eventually produce new rehabilitative strategies.

These approaches have been studied and compared by many researchers and, although there is not full consensus, the following general conclusions can be drawn:

• Regarding neurophysiological and motor learning techniques, there is insufficient evidence to state that one approach is more effective in promoting gait recovery after stroke than any other approach. Furthermore, none of the methods is specifically focused on gait rehabilitation [42,50].

• There is moderate evidence of improvement in walking and motor recovery using robotic devices including systems for BWSTT when compared to conventional therapy [96,91,105-107].

• The combination of different rehabilitation strategies seems to be more effective than over-ground gait training alone.

• Robotic devices need further research to show their suitability for walking training and their effects on over-ground gait [117].

• The use of FES combined with different walking retraining strategies has been shown to result in improvements in hemiplegic gait [121,123-126,207,208].

• Reports on EEG-based BCIs for stroke recovery are limited to the rehabilitation of upper limbs, specifically of hand movements. Moreover, only a few of them have shown a real effect of BCI usage on motor recovery [162,166,171,176,209].

• There is enough evidence to support the assumption that BCIs could improve motor recovery after stroke, but there are no long term and group studies that show a clear clinical relevance.

• Lower limbs and gait function have not been studied in combination with BCIs yet. However some works suggest that there is a common mechanism influencing upper and lower limb recovery simultaneously, independently of the limb chosen for the rehabilitation therapy [164,181].

• Despite the inherent latency of the hemodynamic response fNIRS enables researchers to detect signals from specific regions of the cortex during the performance of motor activities for the development of future BCIs [188-190,195].

• Future research would make possible the analysis of the impact of rehabilitation on brain plasticity in order to adapt treatment resources to meet the needs of each patient and optimize the recovery process.

## Competing interests

The authors declare that they have no competing interests.

## Authors' contributions

JB structured the paper contents and made the revision of the whole article. SM wrote the draft of the manuscript and revised the integration of the different sections. IB provided the framework for the introduction. JM contributed to the robotic devices section, especially exoskeletons and JP revised it and looked for the consistency of this part in the whole paper. DF revised the paper for consistency in the neurophysiological aspects. MM and FT wrote the neurophysiology of gait and classic gait rehabilitation techniques sections. MI revised the manuscript for consistency in the gait rehabilitation approach. AR and AC wrote about fNIRS based BCIs and MR revised it. TS and CB wrote and revised the section of EEG based BCIs. All authors read and approved the final manuscript.
